# General Needs Assessment of the Undergraduate Medical Students to Integrate Courses on Medical Ethics, Time Management and Communication Skills into the Bachelor of Medicine, Bachelor of Surgery Curriculum of Pakistani Medical Colleges

**DOI:** 10.7759/cureus.4433

**Published:** 2019-04-11

**Authors:** Arslaan Javaeed

**Affiliations:** 1 Pathology, Poonch Medical College, Rawalakot, PAK

**Keywords:** medical ethics, communication skills, time management skills, undergraduate medical students, curriculum

## Abstract

In Pakistan, medical ethics, time management, and communication skills are not taught to medical students and are not a part of the curriculum of Bachelor of Medicine, Bachelor of Surgery (MBBS) developed jointly by Higher Education Commission and the Pakistan Medical & Dental Council. The objective of this general needs assessment was to assess the significance of these courses for undergraduate medical students. The literature review of the two online databases (PubMed and ERIC (Education Resources Information Centre)) was conducted in January 2018. The current literature emphasizes the significance of these undergraduate medical courses for medical students in Pakistan.

## Introduction and background

The development of a new curriculum or amending the existing curriculum requires the curriculum developers to establish the need for it. This need is demonstrated by identifying a learning gap that can be defined as a gap between what a student has learnt and what they are expected to learn at a certain point in their education. The proven six-step approach to curriculum development proposed by Thomas, Kern, Hughes, and Chen includes the following: 

Step 1: Problem identification and general needs assessment

Step 2: Targeted needs assessment

Step 3: Goals and objectives

Step 4: Educational strategies

Step 5: Implementation

Step 6: Evaluation and feedback [1].

Problem identification and general needs assessment is the first and foremost step, which leads to a targeted needs assessment. General needs assessment is a process of identifying what already exists and what is missing in the programmes, gaps in services, or curriculum, whereas a targeted needs’ assessment is a process by which curricula developers apply the knowledge learned from the general needs’ assessment to their particular students and learning environment. The purpose of this paper was to understand and identify the general needs assessment of undergraduate medical students to integrate courses on medical education into the undergraduate medical curriculum. Needs assessment not only gives the developers an understanding of the learning gap but also guides about the best way to address it.

Over the last decade, health professionals’ education has been widely recognized as an important specialty in medical schools in Pakistan. The Pakistan Medical and Dental Council (PMDC) has directed all the medical schools to establish a Department of Medical Education (DME) to ensure that quality education is being imparted. The department reviews the content being taught and focuses on effective strategies through which medical students are educated and trained for future challenges. It also concentrates on research and the professional development of the faculty. The significance of making health professionals’ education compulsory for medical teachers is widely emphasized in the literature but, ironically, no significant steps have been taken by the PMDC to implement the same [2]. Apart from clinical knowledge, it is expected that a fresh medical graduate will be equipped with the skills of time management, effective communication, and knowledge of medical ethics, but these competency areas are not part of traditional medical curricula. These three major learning gaps identified will be addressed separately in detail. The PMDC needs to direct its efforts towards forming structured courses designed for the undergraduate curriculum to equip the students with these skills. 

The key terms used in this needs’ assessment are defined as follows:

*1) Undergraduate Medical Curriculum:* The content, teaching and learning strategies, assessment, and evaluation processes exercised throughout five years of training a medical student to graduate as a physician.

*2) Department of Medical Education: * A department that is concerned with promoting excellence in the education of medical students from their entry into the medical school to the point of graduation and thus the contribution to public health. 

*3) Communication Skills*: The capability of conveying ideas and feelings effectively through both verbal and non-verbal means of communication.

*4) Medical Ethics:* The moral principles underlying the practice of medicine.

*5) Time Management skills: *The ability of a student to utilize time effectively by planning and implementing mindful control to increase efficiency and productivity.

Communication skills

Three basic components of effective communication are verbal, non-verbal, and para-verbal components. The verbal component of communication deals with the word selection for the message content. Body language, posture, facial expression, gesture, and spatial distance constitute the non-verbal component of communication. The para-verbal component of communication includes volume, pitch, pacing, and tone of the voice. During a communication, the focus is mostly on verbal components, which only constitutes 10% of the message and the remaining 90% of the message is delivered through non-verbal and para-verbal ways, which are often ignored [3]. The verbal component holds high significance as it includes medical history taking, consent for procedures, conveying difficult information, discussing treatment options, and breaking bad news, etc. Research shows that the way the message is delivered (non-verbal and para-verbal components) greatly influences important factors such as a patient’s compliance to the treatment and patient’s satisfaction level, etc. [3]. The ability to communicate effectively is critical to practicing medicine and fostering this competence is of paramount importance in medical education [4].

Medical ethics

Medical ethics help medical students and doctors in deciding the right course of action from the available choices. It helps in recognising ethical issues that may arise during patient care such as decision-making, clinical judgement, protection of patient’s privacy and confidentiality, breaking bad news, taking informed consent, use of social media, religious preferences, cultural choices, gift acceptance, management of challenging patients and their family members, resource allocation, research with human subjects, and resolving any conflicts of interest. In short, medical ethics provide a doctor with the guiding principles of practicing medicine [5].

Time management

Time management is also an important skill for medical students. When they enter medical school, they need to manage time effectively to avoid stress and ensure success. Time is considered as the greatest asset and in modern-day medicine where enormous learning resources are available, time management becomes a challenge for medical students. The right approach to time management enables students to improve their study-life balance enormously by making the right choices from the onset, such as how to prioritize their tasks and not allowing politeness to overburden them with other students’ work [6].

Guiding questions

This needs assessment is designed to explore the following four questions:

1. Has there been an earlier publication of needs assessment to integrate courses on time-management, communication skills, and medical ethics into the undergraduate medical curriculum? 

2. Do the descriptions, regarding time management, communication skills, and medical ethics as needed by undergraduate medical students, already exist?

3. What challenges exist in incorporating these courses in an undergraduate medical curriculum?

4. What existing material will be beneficial in integrating these courses in an undergraduate medical curriculum for medical students?

These four questions form the backbone of this needs assessment and inform the key search terms explained in the following Methods section.

## Review

Material and methods

A literature review was carried out for this needs assessment. The data for this paper were sourced from PubMed and ERIC (Education Resources Information Centre). Search techniques included Boolean technique and Boolean operator 'AND' was employed. The MeSH (Medical Subject Headings) feature was used during the search in PubMed. To create a more specific search, speech marks were used to group words into specific phrases during the search on ERIC. The terms and their combinations used to search PubMed are listed here: medical education skills AND medical students, communication skills AND medical students, time management AND medical students, medical ethics AND medical students. The search results from PubMed are summarized in Table 1.

**Table 1:** PubMed search results

Search Terms	Results	Relevant Findings
Medical Education Skills AND Medical Students	20	Third-year medical students’ perceptions toward learning communication skills: implications for medical education. Attitudes of freshman medical students towards education in communication skills. Medical students' communication skills in clinical education: Results from a cohort study.
Communication Skills AND Medical Students	20	Teaching communication skills to undergraduate medical students in China. Teaching communications skills to medical students: Introducing the fine art of medical practice. Teaching Communication Skills to Medical and Pharmacy Students Through a Blended Learning Course. Enhancing medical students' communication skills: development and evaluation of an undergraduate training programme Evaluation of a communication skills' training course for medical students using peer role-playing Communication skills of medical students during the OSCE: Gender-specific differences in a longitudinal trend study. Integrating 360° behavior-orientated feedback in communication skills training for medical undergraduates: concept, acceptance, and students' self-ratings of communication competence. Attitudes of medical students toward communication skills learning in Western Saudi Arabia. Assessing Communication Skills of Medical Students in Objective Structured Clinical Examinations (OSCE)--A Systematic Review of Rating Scales. Teaching Inpatient Communication Skills to Medical Students: An Innovative Strategy.
Time Management AND Medical Students	02	Time and Life Management for Medical Students and Residents. Exercise behavior, sleep habits, and time management among students of the Medical University of Lublin.
Medical Ethics AND Medical Students	20	Medical ethics course for undergraduate medical students: a needs assessment study. Knowledge, attitudes, and practices related to healthcare ethics among medical and dental postgraduate students in southern India. Telemedicine as an ethics teaching tool for medical students within the nephrology curriculum. Establishing the need and identifying goals for a curriculum in medical business ethics: a survey of students and residents at two medical centers in Missouri. Medical ethics as practiced by students, nurses and faculty members in Shiraz University of Medical Sciences, Pakistan. Learning objectives achievement in ethics education for medical school students How medical students learn ethics: an online log of their learning experiences. Assessing the Awareness of Egyptian Medical Students about Responsible Conduct of Research and Research Ethics: Impact of an Educational Campaign. The perception of ethics from the point of view of medical students Students' medical ethics rounds: a combinatorial programme for medical ethics education. Integrating Second-Year Medical Students and First-Year Physician Assistant Students Into a 12-Week Ethics Course. Must we remain blind to undergraduate medical ethics education in Africa? A cross-sectional study of Nigerian medical students. The impact of an interprofessional problem-based learning curriculum of clinical ethics on medical and nursing students' attitudes and the ability of interprofessional collaboration: a pilot study. Rural Ethics Ward Rounds: enhancing medical students' ethical awareness in rural medicine. Effectiveness of Course of Medical Ethics for Undergraduate Medical Students.

As ERIC is not a medical education database therefore, unlike PubMed, phrases were used instead of the Boolean operators. The phrases and their combinations used to search ERIC are listed here; “medical education skills and medical education”, “teaching communication skills to medical students”, “teaching time management to medical students" and “teaching medical ethics to medical students”. The search results from ERIC are summarized in Table 2.

**Table 2:** Education Resources Information Centre (ERIC) search results

Search Terms	Results	Relevant Findings
"Medical Education Skills and Medical Education"	0	None
"Teaching Communication Skills to Medical Students"	03	Teaching Communication Skills to First-Year Medical Students. Teaching Communication and Listening Skills to Medical Students Using Life Review with Older Adults. Medical Communication as Art--An Interview with Christine Borland
"Teaching Time Management to Medical Students"	0	0
“Teaching Medical Ethics to Medical Students”	01	Teaching Medical Ethics to Medical Students

Results

After searching the databases and reviewing relevant research papers, it can be said that these medical education courses have not been formally integrated into the undergraduate medical curricula. Research shows that several communication deficiencies are evident in final-year medical students. The research emphasizes the significance of the need for communication skills courses to ensure that every medical student graduates with a required level of communication skills’ proficiency [7]. Communication skills help in determining patient needs and also enable medical students to converse effectively with their colleagues. These are learnt over time with practice and should be incorporated into the medical curriculum [8]. Literature reveals that a communication skills course cultivates these skills in medical students and helps them in acquiring the required competence level [9]. The researchers highlighted in their research that medical students have exhibited a positive attitude towards learning communication skills [10]. After studying these subjects and learning these skills as a part of curriculum, the students will be in a better position to deal with challenging patient situations.

Research also shows that medical students need to manage time effectively to ensure success, but no formal course on this subject is included in the curricula. When students enter a medical school, they cannot manage their time effectively which can cause stress and may lead to failure in examinations. Time management is another course, which needs to be incorporated into the undergraduate medical curriculum to ensure the well-being of medical students by saving them from unnecessary stress. 

The search on PubMed revealed that medical ethics is an important part of practicing the field of medicine. In the United Kingdom, medical ethics is taught as a subject to medical students and this course is an integral part of the undergraduate curriculum. This course enables future doctors to behave according to the principles of ethics. In a study, the courses on medical ethics were introduced which were found as beneficial by the students [11]. Social media has taken our world by storm and physicians face ethical issues concerning professionalism and doctor-patient relationship through these platforms [12]. Medical students would have a better understanding of these issues if they have prior knowledge about medical ethics and would be able to deal with them effectively.

Discussion

In this section, the questions, which formed the backbone of the needs assessment, will be revisited and discussed under the light of the literature review.

1. Has there been an earlier publication of needs assessment to integrate courses on time management, communication skills, and medical ethics into the undergraduate medical curriculum?

Literature review revealed that communication skills and time management are commonly taught through workshops. There are no formal courses on these important subjects, which medical students direly need to become a good doctor. Through this review, it was seen that needs assessment has not been carried out in Pakistan to incorporate these courses formally into the undergraduate medical curriculum.

2. Do the descriptions, regarding time management, communication skills, and medical ethics as needed by undergraduate medical students, already exist?

The description of the needs of these courses is widely available in the literature. Medical ethics is formally taught in some schools, whereas time management and communication skills, despite their need by the students, are not formally integrated in the curriculum. 

3. What challenges exist in incorporating medical education courses in the undergraduate medical curriculum?

The PMDC has directed all the medical schools in Pakistan to establish departments of medical education. These departments are merely a symbol to fulfill the requirement of the council. These departments are playing a limited role in improving medical education in Pakistan. One of the challenges is a shortage of faculty in this field but even in those medical schools where the concerned faculty and specialists are present, no improvement is witnessed. The will to improve appears to be the biggest challenge, and then the shortage of faculty.

4. What existing material will be beneficial in integrating these medical education courses in the undergraduate medical curriculum for medical students?

The already established medical education departments in medical schools across the country can conduct workshops or courses on medical ethics, communication skills, and time management to fulfill the needs of the medical students. PubMed and ERIC databases searches revealed extensive evidence supporting the needs of medical students for these courses. 

## Conclusions

The importance of teaching communication skills, medical ethics and time management is evident through the literature review. In Pakistan, these courses are not taught to medical students and are not a part of the curriculum developed jointly by the Higher Education Commission and the Pakistan Medical & Dental Council. The general needs assessment emphasizes the significance of incorporating these subjects within the medical curriculum. Following the six-step approach to curriculum development, the next step after general needs assessment is the targeted needs assessment. Targeted needs assessment requires systematic data collection through focus groups interviews and surveys of all the stakeholders. These surveys will help in analyzing the knowledge gaps and the preferred mode of teaching such as problem-based learning (PBL), assignments or interactive lectures, etc. The medical education faculty will develop the learning objectives, teaching goals and assessment strategies, and course evaluation, to ensure that students will acquire the desired competency level. These changes will address the learning gaps in medical ethics, communication skills, and time management.
